# *Brassicaceae*-Derived Anticancer Agents: Towards a Green Approach to Beat Cancer

**DOI:** 10.3390/nu12030868

**Published:** 2020-03-24

**Authors:** Luigi Mandrich, Emilia Caputo

**Affiliations:** 1 Research Institute on Terrestrial Ecosystems-IRET-CNR, Via Pietro Castellino, 111, I-80131 Naples, Italy; luigi.mandrich@cnr.it; 2 Institute of Genetics and Biophysics (I.G.B.) “A. Buzzati-Traverso”, CNR, Via Pietro Castellino, 111, I-80131 Naples, Italy

**Keywords:** *Brassicaceae*, plant metabolites, cancer drugs, microbiota, dietary agents

## Abstract

Cancer is the main cause of mortality and morbidity worldwide. Although a large variety of therapeutic approaches have been developed and translated into clinical protocols, the toxic side effects of cancer treatments negatively impact patients, allowing cancer to grow. Brassica metabolites are emerging as new weapons for anti-cancer therapeutics. The beneficial role of the consumption of brassica vegetables, the most-used vegetables in the Mediterranean diet, particularly broccoli, in the prevention of chronic diseases, including cardiovascular diseases, diabetes, and obesity, has been well-documented. In this review, we discuss the anti-tumor effects of the bioactive compounds from Brassica vegetables with regard to the compounds and types of cancer against which they show activity, providing current knowledge on the anti-cancer effects of Brassica metabolites against major types of tumors. In addition, we discuss the impacts of industrial and domestic processing on the compounds’ functional properties before their consumption as well as the main strategies used to increase the content of health-promoting metabolites in Brassica plants through biofortification. Finally, the impacts of microbiota on the compounds’ bioactivity are considered. This information will be helpful for the further development of efficacious anti-cancer drugs.

## 1. Introduction

A large variety of tumors affect the human population. Cancer is characterized by a deregulation of key cellular functions, such as growth signaling, anti-apoptotic signaling, gene stability, immune response, and stromal microenvironment regulation [1]. Although prevention, control strategies, and different treatments are available, the number of cancer patients increases each year, as does the mortality rate for this disease [2].

Numerous clinical trials have been developed to investigate potential cancer cures. However, their toxic side effects have had negative impacts on the patients. Radiotherapy and chemotherapy show severe side effects due to their toxicity on healthy cells. Target-based therapy and immunotherapy, although highly specific in cancer targeting, can be applied to a limited target patient range and are very expensive. Furthermore, several kinds of cancer tend to relapse and acquire resistance after treatment. Currently, combined therapies are substituting monotherapy treatments to overcome their limitations [3]. Moreover, significant effort is being made to search for more effective drugs with reduced side effects from new sources.

For millennia, plants have been used in medical cures. Plant metabolites show a wide range of biological functions, such as anti-cancer, anti-analgesic, antimicrobial, and anti-inflammation activities. More than 60% of drugs with anti-cancer activity are derived from plants [4]. Therefore, plant metabolites represent a good source for the development of efficacious therapeutic agents with reduced side effects.

Several studies have reported the chemopreventive and anti-cancer properties of dietary agents. It has been observed that vegetable consumption is associated with a lower risk of developing cancer. Our main interest is vegetables belonging to the *Brassicaceae* family, such as broccoli and cauliflower, as these vegetables are a source of glucosinolates (GLs), from which other biologically active products are derived [5,6]. Unfortunately, the approach of these studies seems to be heterogeneous and not conclusive [6].

In this review, we discuss the potential properties of GLs derived from *Brassicaceae* as chemopreventive and anti-cancer agents, with respect to their absorption and metabolism in the human body.

## 2. Brassicaceae

*Brassicaceae* (*Cruciferae*) is a large plant family including a wide range of vegetables, condiments, forage, and oil seed crops such as broccoli, cauliflower, cabbage, kohl rabi (all cultivars of *Brassica oleracea*), radish (*Raphanus sativus*), mustard (*Sinapis alba*), and rapeseed (*B. napus*), as well as ornamentals (e.g., *Arabis, Aubrieta, Erysimum, Iberis, Lunaria, Matthiola,* and others), comprising several model plants (e.g., *Arabidopsis thaliana, A. suecica, Arabis alpina, Cardamine hirsuta*) [7].

*Brassicaceae*, also known as the “mustard” family, includes approximately 338 genera and 3709 species and is distributed worldwide in all continents except Antarctica. However, the species diversity is not equally distributed, and the most important diversification centers are found in the Irano-Turanian and Mediterranean regions [8,9,10]. Their distribution pattern and several recent studies suggest that this plant family originated and diversified in the eastern Mediterranean (Asia Minor or present-day Turkey) and adjacent regions [11,12].

**Figure 1:**
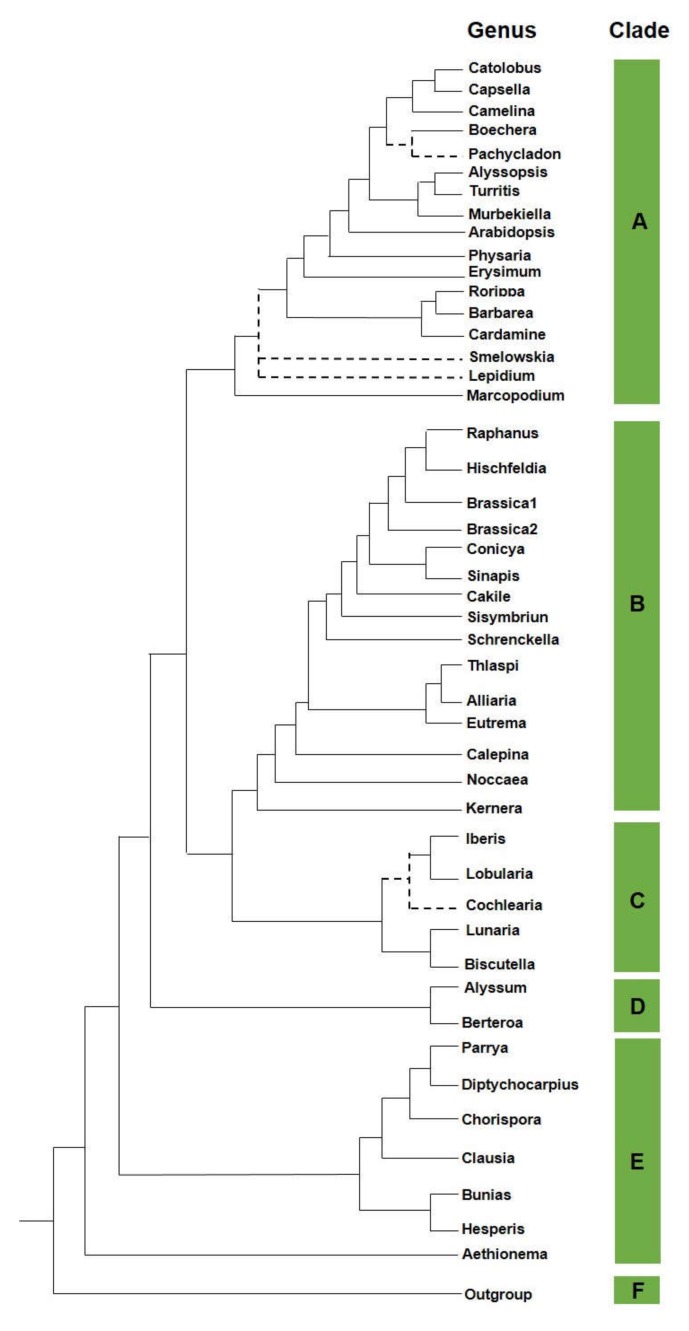
Phylogeny in *Brassicaceae.* A schematic phylogenetic relationship in the *Brassicaceae* family.

The crucifers are phylogenetically sisters to the *Cleomaceae* family, and both families belong to the *Brassicales* order [13,14]. A robust and reliable family tree has been recently developed [15]. It is based on more than 100 orthologous nuclear markers retrieved from sequenced transcriptomes of 55 species belonging to the five major clades within the crown group (Figure 1).

## 3. Brassicaceae Phytochemicals

As with many other vegetables, crucifers are high in minerals, nutrients, and phytochemicals (e.g., selenium, folate, and fiber). Particularly, these vegetables are rich in sulfur-containing compounds, called glucosinolates (GLs), which are responsible for their pungent and spicy taste [16]. GLs are especially abundant among families of the order *Capparales*: *Tovariaceae, Resedaceae, Capparaceae, Moringaceae, and Brassicaceae.* Families outside the order exhibit occasional occurrences and include *Caricaceae, Euphorbiaceae, Gyrotemonaceae, Limnathaceae, Salvadoraceae*, and *Tropaeolaceae* [17].

**Figure 2:**
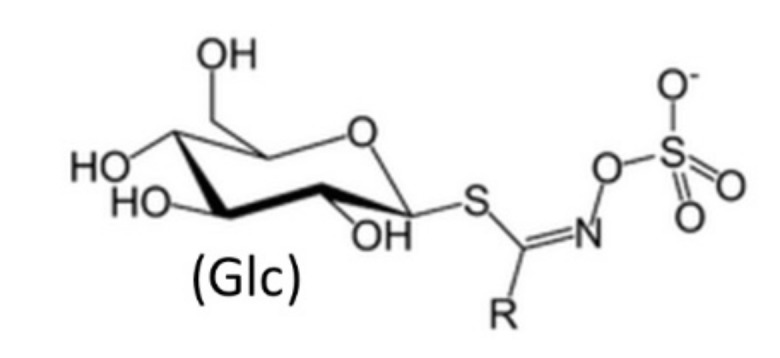
Glucosinolate structure. In the schematic structure of glucosinolate, R is an aliphatic, aromatic, v-methylthioalkyl, or heterocyclic residue.

It has been demonstrated that the chemopreventive potential of cruciferous vegetables is likely due to glucosinolates and their secondary metabolites (e.g., isothiocyanates (ITCs)) [18]. So far, more than 130 different glucosinolates have been identified from various plants, and the profiles of these compounds vary depending on cultivars and growing conditions. Depending on the molecular structure, they can be classified as aliphatic, aromatic, v-methylthioalkyl, or heterocyclic glucosinolates (Figure 2) [16,19,20].

GLs can be hydrolyzed by myrosinase (thioglucoside glucohydrolase, EC 3.2.1.147, formerly EC 3.2.3.1) to produce D-glucose and various other degradation products. Both the enzyme and substrates physically coexist in plants but are separated from each other. In *Arabidopsis thaliana*, GLs have been detected between the phloem and the endoderm, whereas myrosinase is found in the neighboring phloem parenchyma [21]. Further, it has been demonstrated that myrosinase is localized in specialized GLS-free idioblast cells, termed myrosin cells, and its activity is highest in the seed and seedling stages [22,23,24].

**Figure 3:**
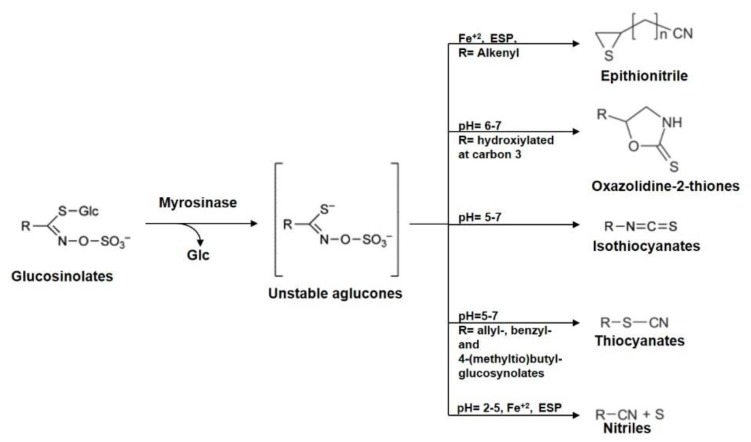
Simplified representation of glucosinolates (GLs) and their hydrolysis products. Myrosinase acts on GLs to form an unstable aglycone intermediate. It can rearrange spontaneously generating an isothiocyanate at neutral pH. Under certain conditions, such as in the presence of nitrile specifier proteins (NSPs), ferrous ions, or at pH < 5, GL hydrolysis is responsible for the formation of the corresponding nitriles. Instead, in the presence of epithiospecifier proteins (ESPs), GL hydrolysis gives epithionitriles from alkenyl GLs through a ferrous ion-dependent mechanism.

Initially, myrosinase-modified GLs release thiohydroximate-O-sulfate, an unstable aglucone (Figure 3). In the presence of the so-called epithiospecifier proteins (ESPs), aglucone degradation leads to the release of epithionitriles (EPTs) from alkenyl GLs [25,26,27] and nitriles from non-alkenyl GLs [28,29,30]. In the absence of ESPs, the aglucones spontaneously rearrange to form isothiocyanates (ITCs) and small amounts of nitriles. Nitrile specifier proteins (NSPs), which favor nitriles, as well as epithiospecifier modifiers (ESMs), which, in turn, favor isothiocyanate formation, have also been identified in *Arabidopsis thaliana* [28,31].

Various studies suggest that GLs may play an important role in herbivore and microbial defense by their potent odor and taste [16]. High levels of these compounds (“mustard oil bomb”) are quickly released upon tissue damage, constituting a very effective defense against some generalist herbivores [32]. Further, GL degradation products are known for their fungicidal, bactericidal, nematocidal, and allelopathic properties [33,34]. Recently, GLs and their degradation products have attracted intense research interest because of their cancer chemoprotective properties.

In plants, the GL profiles and contents are affected by various parameters, including: temperature, irradiation, nutrition, and water supply [35]; their management (organic vs. conventional) [36]; wounding and biotic factors, such as herbivores [37,38]; and by intrinsic plant-determined factors, such as (onto)genetic influences, which can induce an enormous variation in GL levels, even within the plant [39]. Furthermore, according to the stage of plant development, different GL profiles have been found. High concentrations of aliphatic or aromatic GLs have usually been found in seeds [39,40,41], while after germination, their content progressively decreases and the total GL content increases due to higher indole GL levels [39,40,42].

## 4. ITC Anti-Cancer Activity in Different Tumors

In vitro and in vivo studies have shown that ITCs are able to activate phase II detoxification enzymes (such as quinone reductase and glutathione S-transferase) as well as to disrupt tubulin polymerization, inducing cell cycle arrest and the activation of apoptosis in cancer cells [43]. Additionally, since dietary ITCs are well absorbed and have good bioavailability, these compounds are promising candidates for anti-cancer therapies [44,45,46].

Particularly, the bioactivity and fate of the downstream metabolites sulforaphane (SFN) and erucin (ECN) from alkyl isothiocyanates, as well as indole-3-carbinol from indolyl glucosinolates, have been heavily investigated in humans to explore their roles in carcinogenesis and cancer progression modulation (Table 1).

### 4.1. Alkyl Isothiocyanates and Cancer

#### 4.1.1. Sulforaphane

Several studies have shown that SFN induces apoptosis and inhibits the progression and metastasis of many cancers [73,74,75].

Recently, it was found that the consumption of glucoraphanin-rich broccoli soup affects gene expression in the prostates of men on active surveillance, consistent with a reduction in the risk of cancer progression [47]. Broccoli sprouts are considered a functional food as they are naturally enriched in glucoraphanin (GR), which is the biological precursor of sulforaphane (SFN), a compound mainly found in broccoli sprouts, Chinese kale, cabbage, and watercress. Due to its health-promoting value, broccoli sprout juice is becoming very popular.

It was previously reported that dietary consumption of SFN and broccoli was correlated with a reduction in tumor size and growth in a breast cancer rodent model [48]. It has been demonstrated that this effect may be associated with the role of SFN in repressing the cyclin-dependent kinase 4- D-type cyclin (CDK4-CCND) complex via the downregulation of *SERTAD1* gene expression, leading to an increase in the breast cancer population in the G1 phase [49].

Moreover, the anti-cancer effects of SFN and other bioactive phytochemicals from medicinal plants have been investigated in combination with conventional therapies for breast cancer treatment [50]. Interestingly, it has been observed that docetaxel (at a dose of 10 mg/kg once every 7 days) decreased tumor growth of established tumors by 83.2%, whereas SFN (at a dose of 50 mg/kg daily) inhibited primary tumor growth by 37.4% in a SUM149 breast tumor xenograft mouse model. The combination of SFN and docetaxel enhanced the reduction in the primary tumor volume (92.5% reduction compared with control) and synergistically inhibited the cancer stem cell (CSC) population compared with docetaxel alone [51].

Furthermore, preliminary data show that SFN may prevent or treat breast cancer by inducing estrogen-induced metabolic changes [52]. It has been demonstrated that estrogen can enhance purine metabolism to promote DNA biogenesis, while estradiol treatment was shown to induce an increase in amino acids levels, such as L-proline and L-arginine in breast cancer cells (MCF-7). Both purine metabolites and amino acids are critical nutrients for cells, providing them with the necessary energy and cofactors to stimulate their cell survival and proliferation and thus tumor progression. In this context, it has been observed that SFN reduces the levels of related purine metabolites and amino acids in MCF-7 cancer cells, providing a novel insight into the potential mechanism by which SFN may act as a cancer chemopreventive agent.

Additionally, it was demonstrated that sulforaphane is able to reduce the proliferation capacity of human (SKOV3) and mouse (C3 and T3) ovarian cancer cell lines by downregulating cyclin D1 and cyclin-dependent kinases 4 and 6 (CDK4 and CDK6) [55].

Interestingly, sulforaphane has been shown to sensitize cancer cells resistant to chemotherapy. SFN overcame adriamycin- and cisplatin-resistance in ovarian cancer, suggesting that it may help to reduce chemotherapeutic compound doses as well as their side effects [56,57].

The effects of SFN have also been evaluated in melanoma. It has been demonstrated that SFN impairs the viability of melanoma cell lines (A375 and 501MEL cells) in a dose- and time-dependent manner, while it does not affect non-cancerous epidermal melanocytes (HEMa). Studies have shown that SFN increases the percentage of apoptotic melanoma cells by cleaving poly (ADP-ribose) polymerase (PARP), activating p-p53, caspase-3, caspase-8, and caspase-9, and decreasing Bcl2 expression, thus confirming its pro-apoptotic role. SFN is a very attractive multipotent anti-cancer agent, and its use could open new avenues for preventing tumor growth and treating human malignancies [58]. Sulforaphanes as well as other phytocompounds (e.g., curcumin, epigallocatechin gallate (EGCG), resveratrol, lycopene, SR-T100, several flavonoids, wogonin, Wisteria floribunda agglutinin (WFA), ginsenosides, and genistein derivatives) have been demonstrated to reduce CSC-related marker levels and inhibit CSC-related signaling pathways in ovarian cancer, suggesting their potential use to overcome its chemoresistance and relapse, mainly due to the CSC fraction [76].

Interestingly, SFN reverses gefitinib tolerance in human lung cancer cells via the modulation of Sonic Hedgehog (Hh) signaling [77] and regulates the self-renewal of pancreatic CSCs through the Sonic Hh pathway both in vivo and in vitro [78,79]. Additionally, SFN demonstrated a suppressive effect on gastric CSCs via the Sonic Hh pathway [79].

#### 4.1.2. Erucin

Erucin (ERU) is an isothiocyanate derived from the glucosinolate glucoerucin, which is typically found in high amounts in wild rocket (from *Eruca sativa* Mill.).

ERU is also derived from SFN biotransformation by sulfur atom reduction in the human body [80], and has been described as a slow H_2_S-releasing compound [81,82] like other natural ITCs such allyl isothiocyanate, 4-hydroxybenzyl isothiocyanate, benzyl isothiocyanate, and SFN (previously described). H_2_S is a well-known endogenous gasotransmitter, which plays a critical role in the cardiovascular and respiratory systems as well as in cell growth regulation.

Anti-cancer and chemopreventive activities are some of the proposed biological properties described for isothiocyanates. Interestingly, it is their particular behavior known as “hormesis” that has also been used to describe the action of the endogenous gasotransmitter hydrogen sulfide [83,84]. ITCs and low H_2_S concentrations are able to promote cell proliferation, whereas high concentrations inhibit cell proliferation, migration, and angiogenesis in a variety of cancers [84].

It has been described that endogenous H_2_S, as well as exposure to low levels of exogenous H_2_S donors for a short time, lead to anti-apoptotic effects in tumor cells, enhancing cancer growth [84]. On the contrary, long-term treatment with a relatively high concentration of H_2_S donors inhibits tumor cell proliferation in different types of cancers, thus suggesting that slow-releasing H_2_S donors may represent a promising anti-cancer therapeutic strategy [83,84].

ERU is able to increase the release of H_2_S inside AsPC-1 cells, one of the most aggressive pancreatic adenocarcinoma cell lines, in a concentration-dependent manner, exhibiting a hermetic behavior similar to other natural H_2_S donors showing anti-cancer effects [59], such as GYY4137 [60,61] and acetyl deacylase disulfide [62].

Interestingly, ERU, combined with lapatinib, is able to strongly decrease the metastatic potential of breast tumor cells, even the ones with a drug-resistant phenotype [63].

#### 4.1.3. Indole-3-Carbinol in Cancer

Indole-3-carbinol (I3C) is a natural compound derived from the breakdown of the glucosinolate glucobrassicin, which is found in broccoli, cauliflower, cabbage, collard greens, Brussels sprouts, and kale [85].

I3C has shown antiproliferative effects in several colon cancer cell lines (HT29, WS480, Colo320, Caco-2, HCT-116) [64,65,66,67,68] and hepatocellular carcinoma cells [69]. Interestingly, this molecule is able to induce cell cycle arrest by upregulating mi-RNA-34a expression in MCF-7 human breast cancer cells as well as cell death via the apoptosis of ERα-positive breast cancer cells [70,71].

However, most of this evidence refers to in vitro experiments. The process is more complex in vivo. The main critical point is the measurement of these compounds in the organism, for example, isothiocyanates are rapidly metabolized in the gut and liver and are eliminated in urine; furthermore, their half-lives and effectiveness depend on a number of factors, such as how they are absorbed through food, the frequency of absorption, the metabolism of these compounds, as well as genetic factors due to polymorphisms, which can modify the absorption and metabolism of phytochemicals.

## 5. Anti-Cancer Properties of GL Metabolites and Their Bioavailability

As mentioned in the previous section, evidence has shown the potential anti-tumor properties of GL metabolites by using in vitro cell assay, where purified GL metabolites are administrated to cancer cell lines to evaluate the effect of their activity on their survival. However, these assays did not take into account various parameters that affect the bioavailability of these compounds when they are administrated into the diet of humans. For example, the brassica variety, the amount that should be introduced into the diet of cancer patients to be efficacious against tumor progression, and the storage, handling, processing, and cooking of brassica crops to get the maximal bioavailability of their active compounds are some important issues addressed in several investigations.

It was demonstrated that the metabolic content of different GLs depends on the storage, preparation, and cooking processes used. These processes cause the rupture of cellular membranes in the plant leading to contact between GLs and the myrosinase enzyme and then to GL hydrolysis [17,25]. A 5–10-fold change in the concentration of glucosinolates from Brassica vegetables during the post-harvest stages was reported [36,86]. Moreover, previous research demonstrated that partial or total inactivation of myrosinase, breakdown of glucosinolates at high temperatures, loss of enzymatic cofactors, leaching of glucosinolates and their metabolites into the cooking medium or volatilization, and/or thermal degradation of the metabolites can occur during cooking, as simplified in Table 2 [86].

Further, it was observed that environmental, cultivar, and genetic factors as well as industrial processing, storage, and domestic cooking contribute to a change in GL concentrations [25,90]. Additionally, GL content can be affected by the preparation of brassica vegetables before cooking. It was shown that vegetable washing methods after cutting, such as hot/cold water use or different soaking times in water, may enhance the loss of glucosinolates because of their water-solubility properties [91].

Furthermore, when brassica vegetables are ingested, GLs continue to be hydrolyzed by active plant myrosinase in the upper gastrointestinal tract [93] and successively in the colon by the bacterial microflora, which produce the myrosinase enzyme [94,95,96], affecting their bioavailability.

It has been demonstrated that the inter-individual variation in GL hydrolysis is mainly dependent on differences in bacterial microflora between individuals. Bacteria such as *Lactobacillus plantarum* KW30 and *Lactococcus lactis* ssp. lactis KF147 were found to be able to transform 30–33% of glucoraphanin and/or glucoerucin into sulforaphane nitrile, erucin nitrile, and some unknown metabolites. Furthermore, *Lactobacillus agilis* R16, another lactic acid bacterial strain, was reported to be capable of hydrolyzing sinigrin (SNG) into allyl isothiocyanate (AITC) [96]. More microorganisms were also reported to exhibit myrosinase activity, such as *Bacillus cereus* and *Aspergillus niger* [97,98,99,100,101]. Furthermore, the decrease in GL hydrolysis in the gut may depend on bowel microflora reduction due to mechanical cleansing and/or antibiotic treatment.

However, although the colonic microflora is capable of transforming GLs into their active metabolites, the role of plant-derived myrosinase was shown as critical for maximal ITC production in the gut. This finding is supported by a study where a lower bioavailability and delayed appearance of ITC metabolites were observed in the biological fluids (plasma and urine) of individuals consuming a broccoli supplement lacking myrosinase versus those consuming fresh broccoli sprouts. These differences may partially be explained by the fact that plant- and aphid-derived, but not bacterial-derived, myrosinases belong to the glycoside hydrolase (GH) family GH1 and exhibit differences in their activity [102,103].

In humans, the absorption of isothiocyanates occurs in the intestinal epithelial cells, followed by release into the systemic circulation and successive metabolism in the liver through the mercapturic acid (MA) pathway. In particular, ITCs form conjugates with glutathione and are successively subjected to enzymatic modification and are released in urine as *N*-acetylcysteine conjugates or MA products [104,105]. The measurement of the latter (MA) in urine has been used to provide the overall ITC uptake after Brassica consumption [106,107,108,109,110,111].

After brassica ingestion, GL hydrolysis and ITC absorption are influenced by the residual glucosinolate concentrations, as well as the plant myrosinase activity and the composition of the gut microflora and genotypic variation [93,108,112]. Additionally, the fate of glucosinolates is affected by the time they are in the gastrointestinal tract, their delivery to the small intestine for hydrolysis and/or absorption, and the extent of colonic fermentation [113]. Moreover, individuals may show different GL metabolism as a result of polymorphisms in genes coding for glutathione-S-transferases (GST). It plays a key role in isothiocyanate conjugation with glutathione to different extents, which is critical before the excretion of isothiocyanates as MA molecules [112].

Therefore, the quantities of available active ITCs after the consumption of cooked cruciferous vegetables may differ depending on inter-individual differences in the gut microflora due to genetics, environment, or diseases. In particular, differences in the intestinal flora of patients should be evaluated when pre-clinical trials involving cruciferous vegetables are designed. Different types of broccoli supplements containing broccoli and microflora-derived myrosinase may be explored for the development of novel anti-cancer therapeutic strategies based on brassica-derived agents.

## 6. Conclusions

Several studies have shown a correlation between nutrition and cancer risk, although homogeneous data are missing. It was reported that frequent consumption of fruits and vegetables is correlated with better clinical outcomes in ovarian cancer patients after surgery [114], as well as with a reduction in the risk for bladder cancer when combined with milk/yogurt consumption. This is in contrast with the high risk associated with meat consumption, which is related more to the cooking preparation than to the kind of meat [115].

Interestingly, many benefits such as reduced risk of cardiovascular disease, diabetes, and different types of cancer, like prostate, gastric, colorectal, liver, and breast, have been recorded in populations that follow the Mediterranean diet, which is rich in vegetables, legumes, fruits, whole grains, and fish and olive oil as fat sources, and involves moderate consumption of eggs and meat [116].

Further, research demonstrated that the consumption of broccoli, cauliflower, cabbage, or rocket salad is associated with a lower risk of developing cancer. We reviewed the chemopreventive and anti-cancer properties of *Brassicaceae* dietary agents and their pharmacological potential as remedies in cancer therapy. In particular, we focused on glucosinolates as a good source of active metabolites, which could be used for the development of efficacious therapeutic strategies with reduced side effects.

Since 2007, epidemiological studies on the association between the consumption of cruciferous vegetables and cancer risk have been carried out [117]. A lower risk of colorectal, cervical, and lung cancers was found to be associated with a high intake of cruciferous vegetables [117]. Recently, the consumption of cruciferous vegetables has been associated with a low breast cancer risk in Chinese women [118]. Although the data show an evident correlation between cruciferous vegetable consumption and low cancer risk, this study did not take into account the entire diet followed by Chinese women, which is different from those consumed by women in other countries, as are the cooking procedures for vegetables.

Although very promising, the variation in glucosinolate concentration and myrosinase activity within and between Brassica species as well as the microbiota influence are sources of variability in active metabolites present in these foods and can partly explain the weak relationship between Brassica consumption and preventive effects against cancer in men, which were observed in cohort studies. Interestingly, a recent study on the bioavailability of glucoraphanin and SFN from broccoli with different genotypes demonstrated that a broccoli variety with a high glucoraphanin content (*Brassica villosa* with the Myb28 *V*/*V* genotype), when delivered as soup to humans, resulted in enhanced SFN plasma levels, compared to the same variety with a low glucoraphanin content, suggesting its health benefits [118].

More studies including heterogeneous sets of populations and focused on the follow up of cancer patients during their treatment in association with increased use of cruciferous vegetables in the diet, as well as studies on the bioavailability properties of active brassica compounds, are necessary.

## Figures and Tables

**Table 1:** Active metabolites from glucosinolates and their roles in more frequent cancer types (AKT, protein kinase B; PTEN, phosphatase and tensin homolog).

Active Metabolite	Type of Cancer	Function	Glucosinolate/ Crops or Species	Ref.
Sulforaphane (SFN) 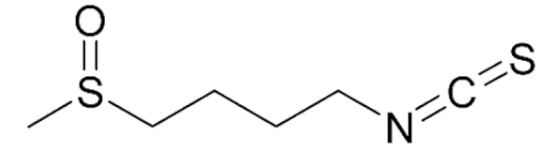	Prostate Cancer	Tumor growth inhibition	Glucoraphanin/Broccoli	[47]
	Breast Cancer	Cell Cycle Inhibition; Sensitize resistant tumor cells to chemotherapy; tumor growth inhibition via CSCs self-renewal regulation		[48,49,50,51,52,53,54]
	Ovarian Cancer	Downregulation of CyclinD1; apoptosis induction by AKT and PI3K pathways modulation.		[55,56,57]
	Melanoma	Tumor growth inhibition via CSCs self-renewal regulation.		[58]
Erucin 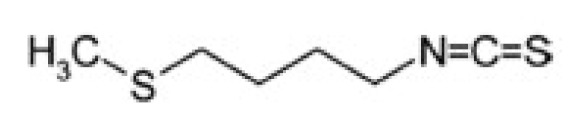	Pancreatic tumor	Tumor growth and migration inhibition	Glucoerucin/Arugula (*Eruca sativa*)	[59]
	Hepatocellular carcinoma	Tumor growth inhibition		[60,61,62]
	Breast cancer	Inhibition of metastasis		[63]
Indole-3-carbinol 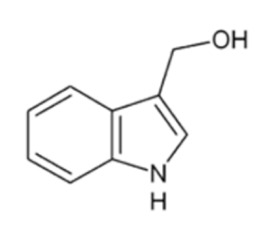	Colon cancer	Apoptosis induction	Glucobrassicin/ Broccoli, cabbage, kale, Brussels sprouts, cauliflower	[64,65,66,67,68]
	Hepatocellular carcinoma	Modulation of mi-RNA-21 expression		[69]
	Breast cancer	Growth inhibion of tumorspheres in vitro and of tumor xenografts in vivo; modulation of mi-RNA-34a expression.		[70,71,72]
	Prostate cancer	PTEN reactivation		[73]

**Table 2:** Factors influencing GL content. Storage conditions and cooking are the main factors involved in the loss of GLs. Here are the reported cases of broccoli; however, different species of *Brassicaceae* are affected in different modes with respect to broccoli *.

	**Storage Conditions**					
	closed environment room temperature (5 days)	open environment room temperature (3 days)		polymeric bags room temperature (7 days)		polymeric film 1 °C (7 days)
GL ** decrease	80%	56%		56%		40%
	**Cooking Conditions**					
	hot water washing	high pressure boiling	conventional pressure boiling		steaming	microwave
GL *** decrease	up to 40%	33%	55%		20%	74%

* [87,88]; ** [87,88,89,90,91,92]; *** [91].
